# Intrafraction tumor motion during deep inspiration breath hold pancreatic cancer treatment

**DOI:** 10.1002/acm2.12577

**Published:** 2019-04-01

**Authors:** Chuan Zeng, Weijun Xiong, Xiang Li, Marsha Reyngold, Richard M. Gewanter, John J. Cuaron, Ellen D. Yorke, Tianfang Li

**Affiliations:** ^1^ Memorial Sloan Kettering Cancer Center New York NY USA

**Keywords:** deep inspiration breath hold, intrafraction motion, pancreatic cancer, radiation therapy

## Abstract

**Purpose:**

Beam gating with deep inspiration breath hold (DIBH) has been widely used for motion management in radiotherapy. Normally it relies on some external surrogate for estimating the internal target motion, while the exact internal motion is unknown. In this study, we used the intrafraction motion review (IMR) application to directly track an internal target and characterized the residual motion during DIBH treatment for pancreatic cancer patients through their full treatment courses.

**Methods and Materials:**

Eight patients with pancreatic cancer treated with DIBH volumetric modulated arc therapy in 2017 and 2018 were selected for this study, each with some radiopaque markers (fiducial or surgical clips) implanted near or inside the target. The Varian Real‐time Position Management (RPM) system was used to monitor the breath hold, represented by the anterior‐posterior displacement of an external surrogate, namely reflective markers mounted on a plastic block placed on the patient's abdomen. Before each treatment, a cone beam computed tomography (CBCT) scan under DIBH was acquired for patient setup. For scan and treatment, the breath hold reported by RPM had to lie within a 3 mm window. IMR kV images were taken every 20° or 40° gantry rotation during dose delivery, resulting in over 5000 images for the cohort. The internal markers were manually identified in the IMR images. The residual motion amplitudes of the markers as well as the displacement from their initial positions located in the setup CBCT images were analyzed.

**Results:**

Even though the external markers indicated that the respiratory motion was within 3 mm in DIBH treatment, significant residual internal target motion was observed for some patients. The range of average motion was from 3.4 to 7.9 mm, with standard deviation ranging from 1.2 to 3.5 mm. For all patients, the target residual motions seemed to be random with mean positions around their initial setup positions. Therefore, the absolute target displacement relative to the initial position was small during DIBH treatment, with the mean and the standard deviation 0.6 and 2.9 mm, respectively.

**Conclusions:**

Internal target motion may differ from external surrogate motion in DIBH treatment. Radiographic verification of target position at the beginning and during each fraction is necessary for precise RT delivery. IMR can serve as a useful tool to directly monitor the internal target motion.

## 1 INTRODUCTION

For locally advanced unresectable pancreatic cancer, conventional doses of radiation are not effective to improve long‐term survival, and stereotactic body radiotherapy or hypofractionated ablative radiotherapy (in 15–25 fractions) has shown promising local control with an acceptable rate of adverse events.^1^ For these types of treatment, since the target doses significantly exceed the tolerance of the surrounding normal tissues, proper organ motion management is crucial to avoid severe complications.

There have been many efforts in characterizing pancreatic tumor motion during free breathing.^2^, ^3^, ^4^, ^5^ Gierga et al reported a study of seven patients using fluoroscopy to observe the motion of fiducial clips.^2^ They found the range of average motion in the superior‐inferior (SI) direction was 4.4–12 mm, while the motion in the anterior‐posterior (AP) direction was much smaller, with a range of average values of 2.5–6.9 mm. Feng et al showed that the tumor border motion was much larger than normally expected, based on 17 cine MRI studies.^4^ They reported that the magnitude of motion for pancreatic tumors, though variable, can be as much as 4 cm in SI direction. The motion in AP direction is less (0.3–1 cm), and the lateral motion was negligible.

Several strategies have been developed in radiotherapy to manage respiration‐induced tumor and organ motion, ranging from passive approaches such as internal margin expansion, to active management such as abdominal compression, breath hold, gating, or dynamic tumor tracking.^6^, ^7^, ^8^, ^9^, ^10^, ^11^, ^12^ In this study, we used the deep inspiration breath hold (DIBH) approach to limit the respiratory motion in treating pancreatic cancer. DIBH has been used for many years for motion management in the treatment of breast, lung, and liver cancers.^11^, ^12^, ^13^, ^14^, ^15^ For example, Dawson et al studied the reproducibility of organ position with active breathing control for 8 liver patients, a total of 262 fractions of treatment, by using repeated fluoroscopy taken *before* each fraction of treatment.^12^ Typically, breath hold treatment relies on some sort of surrogate to reflect the internal target motion. The correlation between the external surrogate motion and the internal target motion have been investigated for breast patients by several groups.^14^, ^15^ To the best of our knowledge, the tumor/organ motion management in DIBH pancreatic cancer treatment has not been studied to date. To find the true tumor location during treatment, in this work, we used the intrafraction motion review (IMR) application (Varian Medical Systems, Palo Alto, CA, USA) on a TrueBeam system, which provides simultaneous kV imaging and MV beam delivery.^10^, ^16^ We directly tracked the internal target (represented by radiopaque marker) in the x‐ray images and characterized the residual motion *during* each fraction of DIBH treatment for the entire treatment course, where residual motion was defined as the maximum observed motion amplitude in each fraction. Also analyzed was the displacement vectors from the initial setup position to the positions observed in IMR images.

## 2 METHODS

### 2.A Patients

An institutional review board/privacy board data exemption was approved before the study. Eight patients who were treated for pancreas cancer at our center since 2017 using DIBH were randomly selected for this study (four men, four women, median age 66 yr, range 48–91 yr). Each patient had internal radiopaque markers (fiducials, surgical clips from attempted resection, and/or biliary stents, etc.) inside or near the target area, all of which are referred to as “fiducials” for simplicity. All patients were treated with a dose of 75 Gy to GTV and 45 Gy to areas at risk for microscopic disease in 25 fractions. The volumetric modulated arc therapy (VMAT) technique was used to deliver the treatment on a TrueBeam system, which can acquire kV images at user‐specified intervals during MV beam delivery, that is, the IMR functionality.

### 2.B CT simulation

**Figure 1:**
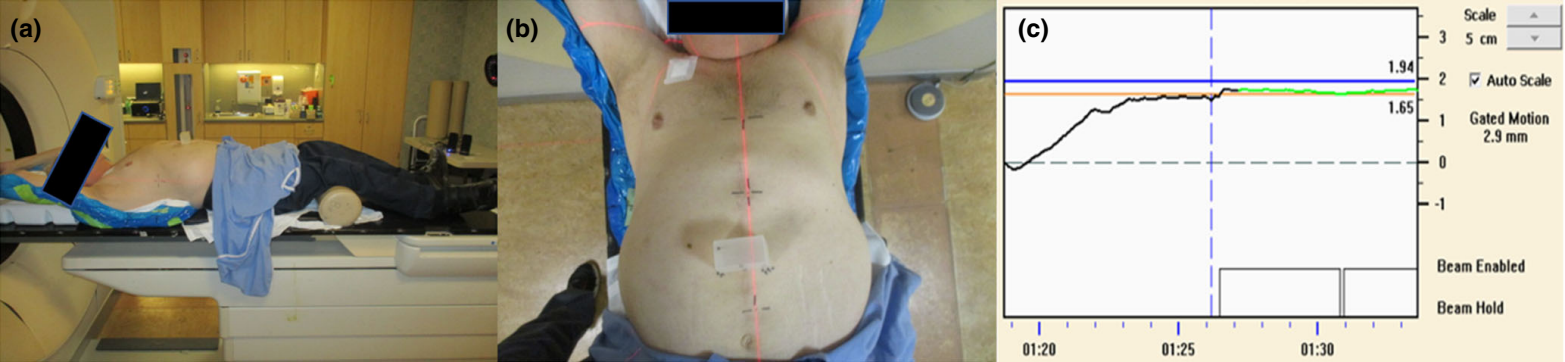
Simulation for deep inspiration breath hold treatment with the Real‐time Position Management system, where the gating window was set to 3 mm. (a) Lateral and (b) anterior view; (c) the graphical user interface showing the gating window.

CT simulation scans were performed by physicians and therapists using a Philips Brilliance Big Bore CT simulator (Philips Medical Systems, Cleveland, OH, USA), with the patient in the supine, arm‐raised position immobilized in a customized mold. The patient's respiration was monitored using the Varian Real‐time Positioning Management (RPM) system (RPM v1.7, Varian Medical Systems, Pala Alto, CA, USA). Patients were selected on the basis of their ability to comply with breath hold instructions and having a reproducible breath hold pattern. Contrast was administered prior to the DIBH CT scan, and a second DIBH scan was often acquired at a later contrast phase; one of the DIBH scans was used for treatment planning depending on the target visibility. A free‐breathing CT scan was also performed as a backup, should the patient become incapable of doing a reliable breath hold later. The patient breathing pattern, that is, the RPM trace, was recorded and imported into TrueBeam to be used as a reference for treatment. Usually an appointment time of 60 min was allotted for simulation to allow for breath hold coaching and practice, assessment. Figure 1 shows a patient during a DIBH CT scan, where an RPM block was placed on the chest, with block position tattooed on the skin for subsequent use in treatment. A typical DIBH RPM trace is also shown in Fig. 1.

### 2.C Treatment planning

CT datasets were transferred to the Eclipse treatment planning system (Varian Medical Systems). The gross tumor volume (GTV) including the tumor and involved regional nodes was outlined on the DIBH CT data by the attending physician. The planning target volume (PTV) was created with average CTV‐to‐PTV margin of 3–5 mm and edited as needed for normal tissue protection. For more details about contouring and the ablative treatment, please refer to Ref. 1. Fiducials were identified during planning process for later comparison. VMAT treatment plans were developed with planning goals to meet the departmental normal tissue constraints while covering the PTV according to the prescription. For most of cases, all planning goals were met with two or three arcs; but more arcs were added when necessary.

### 2.D Treatment delivery and intrafraction imaging

All treatments were delivered on a Varian TrueBeam LINAC with RPM system. Before each treatment, the RPM block was placed at the same position as tattooed at simulation. The patient was coached to maintain DIBH while cone beam CT (CBCT) was taken under a stop‐and‐go acquisition mode to obtain a complete volume dataset under breath hold. The CBCT images were then registered to the planning DIBH CT based on the implanted fiducial markers and couch shifts were made accordingly.

During treatment, the patient was coached to perform DIBH so that the RPM signal was maintained voluntarily in the 3‐mm gating window. If the patient's breath was out of the gating window during treatment, the MV beam was automatically held until the RPM trace came back to the gating window again; typically, this was done by the therapist coaching the patient into a new breath hold. The intra‐treatment kV image acquisitions were triggered every 40° gantry rotation using the IMR function. Each acquired kV image was displayed with graphically overlaid fiducial contours to assist the therapists in evaluating the intrafraction motion. All kV images acquired during each gantry rotation were combined into a movie and automatically saved to an image review system (Offline Review; Varian Medical Systems). For a typical 3‐arc DIBH fraction, about 27 IMR frames can be acquired, resulting in a total of over 600 images for the entire treatment course for each patient.

### 2.E Motion analysis

**Figure 2:**
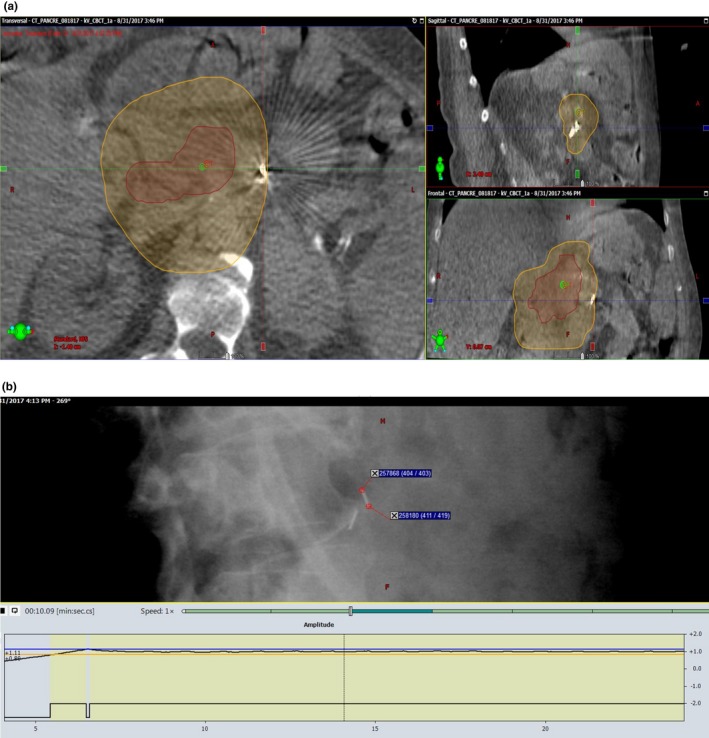
A fiducial marker in the setup CBCT image (a) and its corresponding location in the intrafraction IMR image (b). The contours shown in (a) corresponded to two prescribed dose levels, 45 Gy (orange) and 75 Gy (red), respectively. The pixel information was recorded for both tips of the fiducial in each IMR image.

To track the change of the target location during treatment, we first needed to identify a fiducial marker inside or near the target in the setup CBCT image and record its three‐dimensional room coordinates so that we can compare them with the corresponding fiducial locations found in IMR images. A typical fiducial marker was about 6 mm in length as measured from CBCT. The number of fiducial markers varied from patient to patient. If more than one fiducial marker were available, one was selected and analyzed for each patient according to discernibility and relevance to target. Specifically, the IMR movie was retrospectively reviewed in Offline Review. Each kV image frame from the IMR movie was carefully examined; and the same fiducial marker was identified in each frame. As the gantry rotates during VMAT delivery, the fiducial position changes in the kV images. The pixel positions of the two ends of the fiducial in the two‐dimensional images were manually recorded as shown in Fig. 2.

**Figure 3:**
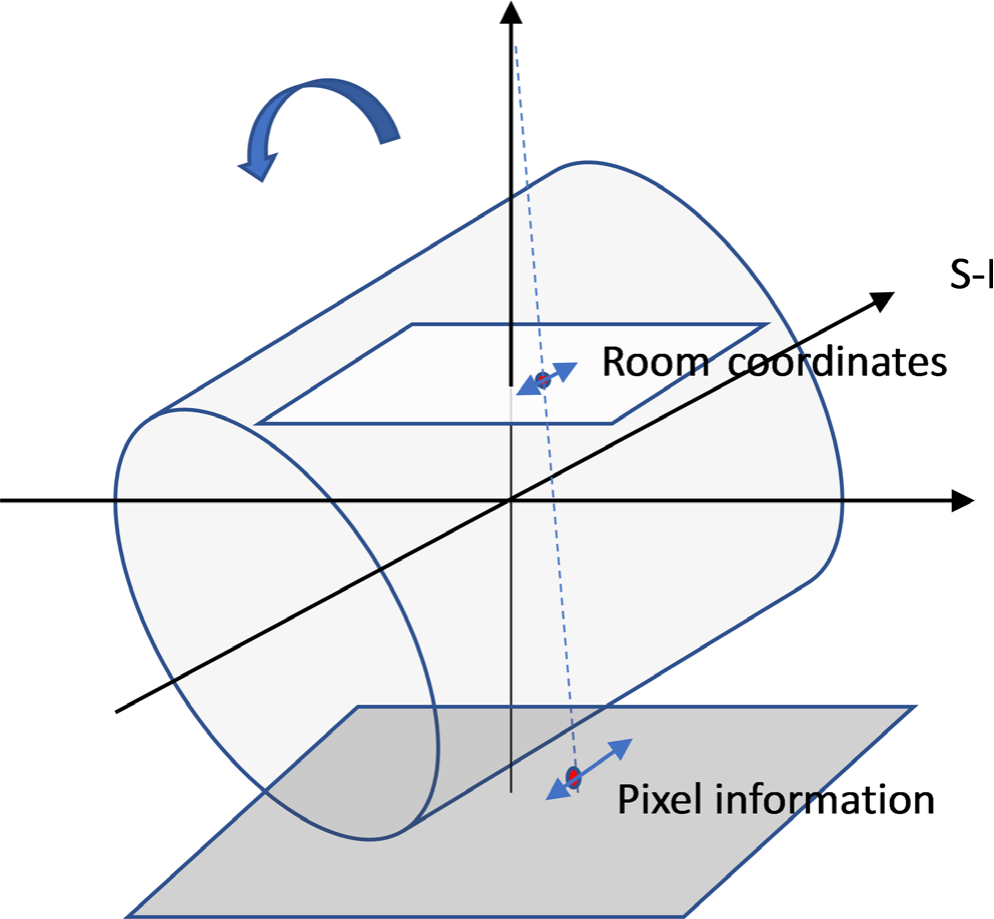
Translate the pixel information of the fiducial on IMR image to room coordinates by back‐projecting the fiducial to the plane where the fiducial was initially localized during setup CBCT.

To eliminate the geometry amplification effect from the x‐ray projection, the pixel information was translated into the room coordinates based on the x‐ray source to the flat‐panel detector geometry. Basically, the fiducial marker in the IMR image was back‐projected to the plane where the fiducial was located in the CBCT image (see Fig. 3). With a single two‐dimensional (2D) IMR image, we can only reliably detect the SI direction motion. However, since the SI motion is usually the largest among all three directions, as reported by other researchers in studying the free breathing pancreatic tumor motion,^2^, ^3^, ^4^, ^5^ we believe, especially under DIBH, it is sufficient to characterize the pancreatic tumor motion with only the fiducial SI coordinates. In this back‐projection calculation, marker coordinates in the AP and lateral directions determined from the CBCT contributed to the magnification factors from their physical locations to the imaging plane. During treatment, motion in the AP or lateral direction may affect the magnification factor and introducing an error proportional to the ratio of motion amplitude to the source‐to‐axis distance (SAD). Since the AP or lateral direction was much smaller than the kV SAD of 100 cm, this error of <1% is negligible in our estimation of SI motion.

## 3 RESULTS AND DISCUSSION

**Figure 4:**
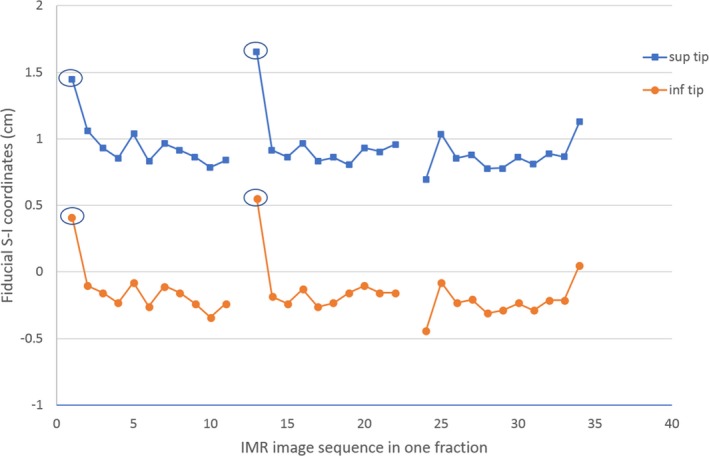
The fiducial coordinates in superior‐inferior dimension during one fraction of deep inspiration breath hold treatment for one patient. The circled data points are the outliers corresponding to the kV images taken outside the gating window. The breaks correspond to intervals between arcs.

Figure 4 shows the position change of a fiducial marker for a patient during one fraction of DIBH treatment. The red and blue dots represent the SI coordinates of the two tips of the fiducial (as illustrated in Fig. 2), respectively. There are two data pairs (circled as outliers) that are more than 5 mm away from the group‐averaged positions, which were from IMR images taken outside of the DIBH gating window. Note that, although the treatment beam was gated by the RPM signal, currently there was no interlock between the TrueBeam IMR imaging system and RPM gating system. Similar outliers have been manually removed throughout the study based on the time points recorded on the RPM gating curve when the simultaneous kV images were taken. It was found that, for this particular patient, the maximum residual motion under DIBH was about 7 mm during the entire treatment course, and less than 5 mm for 15 out of 25 fractions.

**Figure 5:**
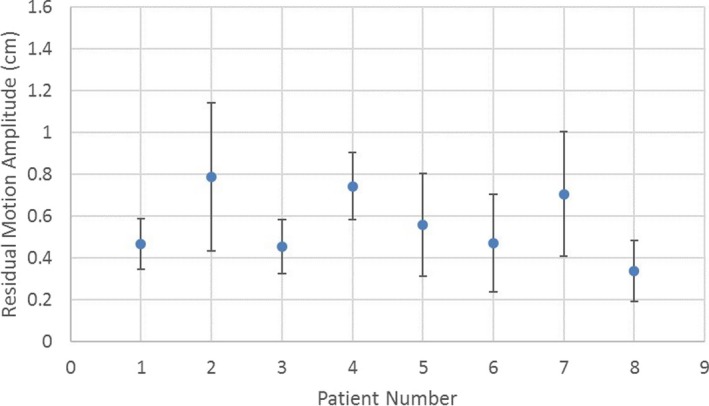
Residual motion for all eight patients over their entire treatment courses.

To quantify the effectiveness of the RPM‐gated DIBH technique in controlling the respiratory motion for the cohort, we first studied how much the target can move under DIBH, that is, the residual motion amplitude. In Fig. 5, we plotted the peak‐to‐peak amplitudes of the residual motion under DIBH for all eight patients, where the center circle represents the mean internal motion amplitude and the error bar shows the standard deviation of the internal motion over the whole treatment course. For four out of the eight patients, the average SI internal motion was within 5 mm; and there were three patients with standard deviation larger than 3 mm, which demonstrated that the internal motion could be larger than the motion shown by the external body marker. Instantaneous residual motion could be as large as >1 cm; but such offset may not persist over the entire session. Under the monitoring using IMR, therapists would intervene if large offset persist over two consecutive kV images.

While the residual motion study reveals the magnitudes of the internal target motion when patients are under DIBH, the displacement of the tumor from its planning position is directly related to the beam targeting accuracy. In this study, the displacement was defined as the difference between the real‐time tumor position found from in‐treatment IMR images and the initial tumor position found from the setup CBCT image, with the fiducial marker representing the tumor location. Table 1 shows the statistics of the displacement for all eight patients over the entire treatment course, where a positive number means a displacement in the superior direction and a negative number means a displacement in inferior direction. The average target position was within 2 mm from the planning position for six out of eight patients; and for all patients the standard deviations were *<*5 mm.

**Table 1:** Displacements of tumor relative to its initial position determined in setup CBCT image

Patient	1	2	3	4	5	6	7	8
Avg (cm)	0.01	−0.30	−0.02	−0.24	−0.09	0.05	0.19	−0.11
Std (cm)	0.22	0.30	0.23	0.36	0.43	0.22	0.39	0.25
Max (cm)	0.87	0.93	0.57	0.81	1.38	0.72	1.38	0.40
Min (cm)	−0.60	−1.17	−0.72	−1.04	−0.95	−0.43	−0.62	−0.64

**Figure 6:**
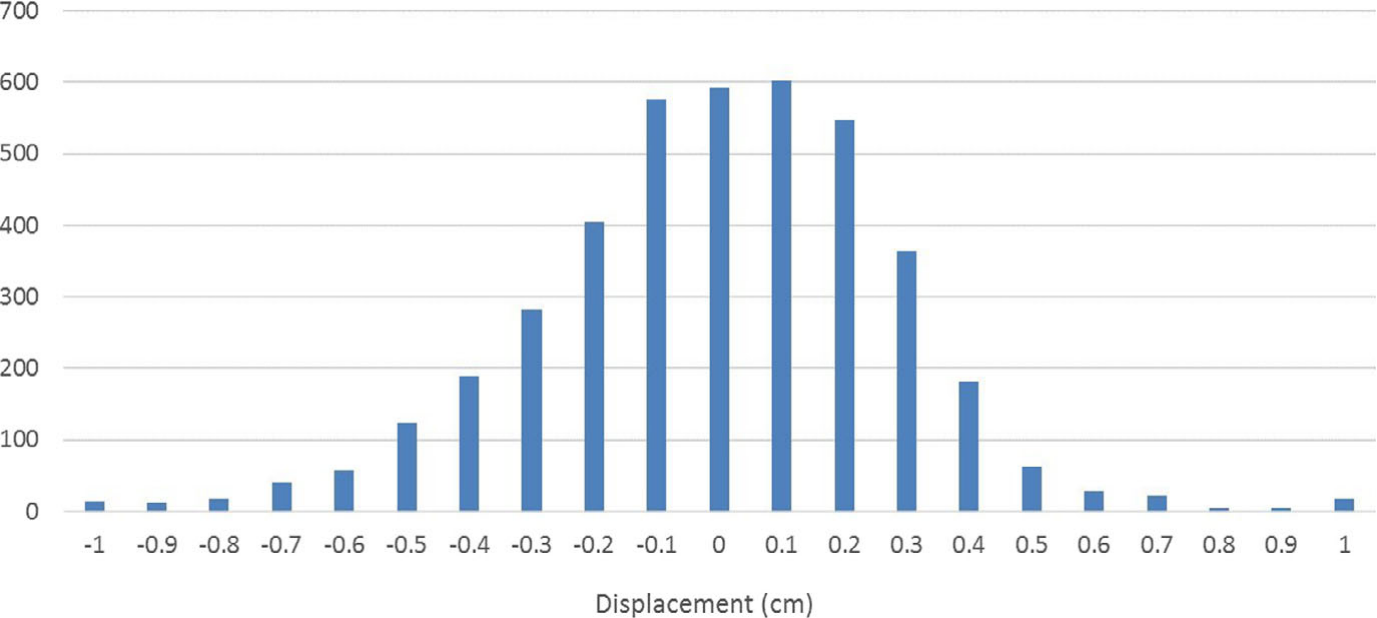
Histogram of the tumor displacements relative to the initial position determined at the beginning of each fraction for all patients.

Figure 6 shows the histogram of the displacements found from over 5000 IMR images of all patients collectively. The mean displacement for the population was −0.6 mm; and the standard deviation was 2.9 mm. For most of the IMR images taken during DIBH treatment, the displacements along SI direction were within 3 mm (78.5%); for about 90.1% cases, the displacements were within 5 mm; and for 0.6% cases, the displacements were more than 1 cm. For a typical patient, high dose PTV *D*_95%_ dropped by about 5% with 3 mm SI shift; >10% with 5 mm shift; and >20% with 1 cm shift. However, the minimum dose to the GTV was maintained above 99% for 3 mm shift and >95% for >5 mm shift.

In this study, we evaluated the internal target motion during DIBH pancreatic cancer treatment. Although the DIBH procedure limited the external marker motion to within 3 mm, the internal motion magnitudes were often found larger, about 5 mm in average, and in some extreme cases could exceed 1 cm. This is consistent with other studies about the correlation between the external surrogate motion and internal tumor motion in general.^13^, ^14^, ^15^, ^17^, ^18^

Furthermore, it was found that the residual motion was typically random rather than a systematic superior or inferior drift. For the population, the average displacement was close to zero, which indicates the mean target position during treatment was very close to the initial position established during CBCT setup. It is likely due to effective immobilization and randomness of the residual motion. The standard deviation of the displacement was found to be 2.9 mm from the eight‐patient full course study. By using van Herk's formula with linear approximation, where the CTV‐to‐PTV margin is given by 2.5Σ + 0.7*σ*,^19^ the extra margin to account for the intrafraction respiratory motion in DIBH treatment is about 2 mm.

## 4 CONCLUSION

Internal target motion may differ from external surrogate motion in DIBH treatment of the pancreatic cancer. IMR serves as a useful tool to directly monitor the internal target motion, with implications in internal margin determination for clinical motion management protocols.

## CONFLICT OF INTEREST

No conflict of interest.
